# Isolation and Mutagenesis of a Capsule-Like Complex (CLC) from *Francisella tularensis*, and Contribution of the CLC to *F. tularensis* Virulence in Mice

**DOI:** 10.1371/journal.pone.0019003

**Published:** 2011-04-22

**Authors:** Aloka B. Bandara, Anna E. Champion, Xiaoshan Wang, Gretchen Berg, Michael A. Apicella, Molly McLendon, Parastoo Azadi, D. Scott Snyder, Thomas J. Inzana

**Affiliations:** 1 Biomedical Sciences and Pathobiology, Virginia-Maryland Regional College of Veterinary Medicine, Virginia Polytechnic Institute and State University, Blacksburg, Virginia, United States of America; 2 Department of Microbiology, University of Iowa, Iowa City, Iowa, United States of America; 3 Complex Carbohydrate Research Center, University of Georgia, Athens, Georgia, United States of America; Universidad Nacional, Costa Rica

## Abstract

**Background:**

*Francisella tularensis* is a category-A select agent and is responsible for tularemia in humans and animals. The surface components of *F. tularensis* that contribute to virulence are not well characterized. An electron-dense capsule has been postulated to be present around *F. tularensis* based primarily on electron microscopy, but this specific antigen has not been isolated or characterized.

**Methods and Findings:**

A capsule-like complex (CLC) was effectively extracted from the cell surface of an *F. tularensis* live vaccine strain (LVS) lacking O-antigen with 0.5% phenol after 10 passages in defined medium broth and growth on defined medium agar for 5 days at 32°C in 7% CO_2_. The large molecular size CLC was extracted by enzyme digestion, ethanol precipitation, and ultracentrifugation, and consisted of glucose, galactose, mannose, and Proteinase K-resistant protein. Quantitative reverse transcriptase PCR showed that expression of genes in a putative polysaccharide locus in the LVS genome (FTL_1432 through FTL_1421) was upregulated when CLC expression was enhanced. Open reading frames FTL_1423 and FLT_1422, which have homology to genes encoding for glycosyl transferases, were deleted by allelic exchange, and the resulting mutant after passage in broth (LVSΔ1423/1422_P10) lacked most or all of the CLC, as determined by electron microscopy, and CLC isolation and analysis. Complementation of LVSΔ1423/1422 and subsequent passage in broth restored CLC expression. LVSΔ1423/1422_P10 was attenuated in BALB/c mice inoculated intranasally (IN) and intraperitoneally with greater than 80 times and 270 times the LVS LD_50_, respectively. Following immunization, mice challenged IN with over 700 times the LD_50_ of LVS remained healthy and asymptomatic.

**Conclusions:**

Our results indicated that the CLC may be a glycoprotein, FTL_1422 and -FTL_1423 were involved in CLC biosynthesis, the CLC contributed to the virulence of *F. tularensis* LVS, and a CLC-deficient mutant of LVS can protect mice against challenge with the parent strain.

## Introduction

*Francisella tularensis* is a Gram-negative coccobacillus, and the etiologic agent of tularemia in a wide variety of animals and humans. *F. tularensis* resides in macrophages, hepatocytes, and a variety of other cells as a facultative intracellular pathogen, but may also be found in the blood during infection [1]. Humans may acquire the agent by handling infected animals, ingesting food or water containing the pathogen, through bites from arthropod vectors (*e.g.* ticks), or by aerosol, which is the route of exposure of most concern due to intentional release of this agent. The most pathogenic isolates of *F. tularensis* are type A1 strains (subspecies *tularensis*), which may cause human infection with as few as 10 organisms [2], [3], and are associated with 30% mortality in the absence of antibiotics following pneumonic tularemia [1], [4]. Type B strains (subspecies *holarctica*) are also highly virulent, but are not associated with the same level of mortality as subspecies *tularensis* [2].

Due to their ease of culture and dispersal, persistence in the environment, and high virulence, *F. tularensis* is classified as a Category-A select agent by the CDC [2]. An approved, licensed vaccine for tularemia is not currently available. However, a live vaccine strain (LVS) was developed in the former Soviet Union from a type B strain following extensive passage and testing *in vitro* and in animals [5]. LVS has been used to protect laboratory workers from infection with type A strains [6], but is not currently approved as a vaccine for the general population due to its poor characterization, potential instability, and questionable safety for immuno-compromised individuals [7]. Although attenuated in humans, LVS is antigenically identical to type A strains, and has been used extensively in research as this strain remains highly virulent for mice, particularly by the intraperitoneal (IP) and respiratory routes [8].

Although *F. tularensis* was first isolated nearly 100 years ago [9], relatively little is known regarding its surface components that contribute to virulence. The lipopolysaccharide (LPS) has been well characterized, and is required for resistance of *F. tularensis* to antibody and complement-mediated bactericidal activity and for virulence [10], [11], [12], [13]. Antibodies to the O-antigen provide protection to mice challenged with LVS [14], [15], but not against challenge with type A strains [16]. LVS mutants lacking O-antigen induce some protection against challenge with LVS or type B strains, but protection against type A challenge is inadequate [11], [12], [13], [17]. Although individual outer membrane proteins have not provided protection against challenge of mice with type A strains [18], a native outer membrane protein preparation did provide partial protection [19].

An electron-dense surface material resembling a capsule has been demonstrated around types A and B strains of *F. tularensis* by electron microscopy (EM), resulting in the conclusion that these subspecies may be encapsulated [20], [21], [22], [23]. Furthermore, a halo-like appearance has been reported around individual *F. tularensis* cells within macrophages [24], [25], and it has been hypothesized that once the bacteria are inside the late endosome/phagosome compartment, certain components of the bacterial capsule or membrane are rapidly released leading to the degradation of the membrane and release of the bacteria into the cytoplasm [26]. However, these electron dense surface structures are not always visible, suggesting this capsule-like complex (CLC) is upregulated under specific environmental/growth conditions [27]. A carbohydrate-protein-lipid component distinct from LPS was identified by Hood that is readily removed under hypertonic conditions [28], and its expression can be enhanced by repeated subculture in defined medium [27]. This crude extract from *F. tularensis* strain SCHU S4 contained carbohydrate (including mannose, rhamnose, and two unidentified dideoxy sugars), as well as amino acids, and −OH 14∶0 and 16∶0 fatty acids. However, a specific component was neither purified nor well characterized. Recently, Apicella *et al*. [29] described an O-antigen capsular polysaccharide around all *F. tularensis* type A and B strains tested. Mutations in genes encoding for O-antigen glycosyltransferases blocked LPS O-antigen and capsule biosynthesis, but mutations in genes encoding for O-antigen polymerase or acyltransferase only prevented LPS O-antigen synthesis, not capsule synthesis. Furthermore, Lindemann et al. [30] identified a locus in strain SCHU S4 separate from the O-antigen locus [31] that is required for LPS O-antigen and/or capsule biosynthesis. Mutations in any of the three genes in this locus resulted in loss of LPS O-antigen and/or O-antigen capsule and increased serum sensitivity of SCHU S4. In addition, the mutants were taken up by human monocyte-derived macrophages more rapidly, but did not continue to increase their replication after 16 hours. Macrophages infected with the mutants also undergo early cell death, in contrast to macrophages infected with SCHU S4. Therefore, the LPS O-antigen and/or the O-antigen capsule are essential to *F. tularensis* persistence in macrophages and replication in the host. The *F. tularensis* genes *capBC* have low-level homology to *capBC* of the *Bacillus anthracis capBCADE* locus, which encodes for proteins that synthesize the poly-D-glutamic acid capsule [32], [33]. Deletion of *capB* in both *F. tularensis* LVS and the highly virulent SCHU S4 strain attenuate the bacteria, which are capable of inducing protection in mice against challenge with the parent strain [34], [35], [36], [37]. However, poly-D-glutamic acid has not been found in any extracts of *F. tularensis* [36], [38], and there is no evidence that *capBC* contributes to synthesis of the electron dense CLC.

A genetic locus that may encode for proteins involved in synthesis and export of a polysaccharide other than LPS has been identified in the genome sequence of *F. tularensis* LVS (NC_007880), and the same genes are present in type A strain SCHU S4 [32] (NC_006570). This locus contains 12 putative genes in the LVS genome: FTL_1432 through FTL_1421. We sought to purify and analyze the electron dense CLC that appears to be upregulated under specific growth conditions, with emphasis on the carbohydrate component. Furthermore, to determine if the above locus is involved in synthesis of the carbohydrate component of the LVS CLC, two putative genes encoding for proteins with homology to a galactosyl transferase and a mannosyl transferase were deleted by allelic exchange. The CLC isolated appeared to be a glycoprotein and distinct from the O-antigen capsular polysaccharide. The glycosyl transferase mutant lacked CLC expression, was attenuated in mice, but provided protection against subsequent challenge with the parent.

## Results

### Extraction of CLC

Gentle extraction of LVS with 10% NaCl [28] following growth on Chocolate agar for several days yielded a carbohydrate that was distinct from LPS, as determined by gas chromatography/mass spectrometry (GC/MS). However, the yield of this material was poor and many other components were present in the extract, including LPS and a wide variety of proteins.

**Figure 1:**
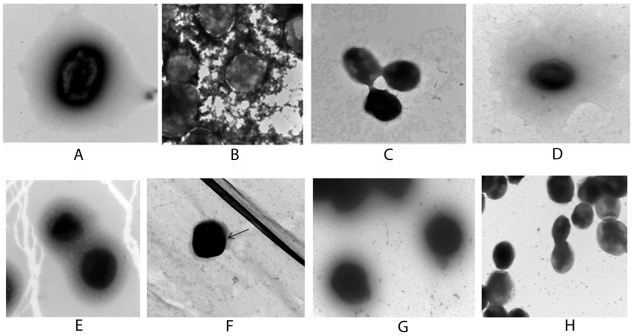
Negative stain electron microscopy of the CLC of *F. tularensis*. Panels A and E: type B strain LVS_P10 grown at 32°C on Glc-CDMA; different cultures were examined on different days; Panel B: type A strain TI0902, passed and grown to enhance CLC expression as for LVS. In this and some other cases the CLC appeared to aggregate, which also occurred following isolation of the CLC; Panel C: glycosyl transferase mutant LVSΔ1423/1422_P10; Panel D: complemented strain LVSΔ1423/1422[1423/1422^+^]_P10; Panel F: LVS not passed in defined medium and grown on CDMA at 37°C; only a small amount of CLC is visible (arrow); Panel G: O-antigen mutant WbtI_G191V__P17 grown as for LVS_P10; Panel H: O-antigen and CLC double mutant WbtI_G191V__P17Δ1423/1422. Strains LVS_P10, WbtI_G191V__P17, and type A strain TI0902_P10 have an electron dense layer surrounding their cells. This layer is missing in mutants LVSΔ1423/1422_P10 and WbtI_G191V__P17Δ1423/1422, and is restored in complemented strain LVSΔ1423/1422[1423/1422^+^]_P10]. The bacteria were fixed in glutaraldehyde, and stained with uranyl acetate. Magnification is 20,000 X, and the scale bar is 500 nm.

Cherwonogrodzky [27] reported that daily passage of *F. tularensis* LVS in Chamberlain's defined medium broth (CDMB), followed by growth on Chamberlain's defined medium agar (CDMA), resulted in an increase of the electron dense material surrounding the bacteria. Therefore, to enhance CLC synthesis and minimize contamination with LPS, the LVS O-antigen mutant WbtI_G191V_ [11] was subcultured daily in CDMB for seventeen days (WbtI_G191V__P17), followed by growth at 32°C for 5 days on CDMA in 7% CO_2_. Parent strain LVS was passed in the same way for 10 days to obtain LVS_P10. Negative staining EM confirmed that such passage resulted in an increase in CLC synthesis around LVS_P10 (Fig. 1A and 1E), and around WbtI_G191V__P17 (Fig. 1G), but very little CLC (arrow) was seen around LVS that had not been passed in CDMB and cultured at 37°C (Fig. 1F). Similar results were obtained with type A strains SCHU S4 and TI0902 that were passed in the same way in CDMB and CDMA (Fig. 1B). In the latter analysis, but also in others, the CLC around the cell appeared to aggregate and was more dense than diffuse, possibly due to fixation during EM (Fig. 1B). After scanning multiple micrographs (>10) of strains enhanced for expression of CLC (LVS_P10, WbtI_G191V__P17, TI0902 passed 10 times, and passed complemented mutant LVSΔ1423/1422_P10) at least 75% of the cells were observed to make at least as much CLC as shown in Fig. 1. In contrast, no cells of either of the CLC mutants (LVSΔ1423/1422_P10 and WbtI_G191V__P17Δ1423/1422) described below were observed to have enhanced CLC around them. Western blot analysis of LPS from exactly the same number of cells of LVS and LVS_P10 showed there was no increase in the amount of LPS on passed cells (data not shown). Therefore, the enhanced electron dense material around the passed cells was not LPS.

We also tested the effect of supplementing CDMA with the carbon sources glycerol, galactose, or glucose to further enhance CLC synthesis. LVS_P10 colonies grown on 1% glycerol appeared similar to LVS grown on CDMA. There was substantially less growth and colony iridescence of bacteria incubated on 1% galactose. However, colonies grown on 1% glucose (Glc-CDMA) appeared more mucoid and more iridescent than those on CDMA alone (data not shown). When the CLC was extracted from 10 plates of WbtI_G191V__P17 grown on each carbon source at 32°C for 5 days there was 10% or more CLC recovered from bacteria grown on Glc-CDMA than on media supplemented with the other carbon sources, as determined by protein and carbohydrate assays (data not shown). Therefore, the bacteria were grown on Glc-CDMA for subsequent extraction of CLC.

When WbtI_G191V__P17 was grown on Glc-CDMA as described above, extracted with 0.5% phenol, and the cells removed by centrifugation, the extract was thick, frothed easily, and was slightly yellow in color. Furthermore, this extract became highly insoluble when the phenol was removed or the material concentrated. The solubility of the extracted CLC was greatly improved after ethanol precipitation, digestion with RNase, DNase, and in particular Proteinase K (which also eliminated frothing). Following ultracentrifugation and dialysis, further purification (primarily to remove ribose) was obtained by gel filtration through Sephacryl S-300 (see Materials and Methods). Approximately 2.8 mg of purified, LPS-free CLC was obtained per gram (wet weight) of WbtI_G191V__P17 cells grown on Glc-CDMA.

### Physical and chemical characterization of the CLC

**Figure 2:**
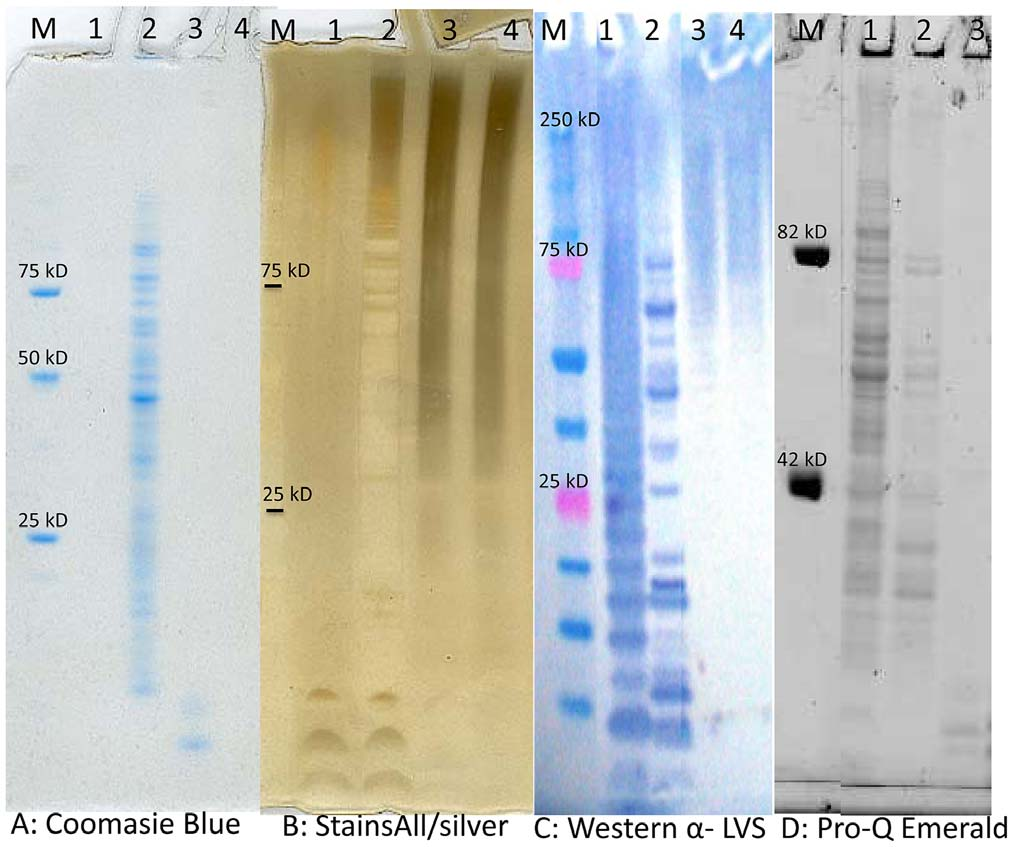
Polyacrylamide gel electrophoresis of CLC extracts. CLC extracts at various stages of purity, or *F. tularensis* LPS as a control, were separated by electrophoresis in a 4–12% separating gel, and the components identified by (A), Coomassie Blue for protein; (B), Stains-All/silver stain for acidic molecules; (C), Western blot with antiserum to LVS whole cells for antigenic components; (D), Pro-Q Emerald stain for carbohydrate. Lanes for panels A-C: M, molecular size standards; 1, LVS LPS (20 µg); 2, crude CLC prior to enzyme digestion (20 µg); 3, CLC extract following enzyme digestion (20 µg); 4, purified CLC (20 µg). Lanes for panel D: M, molecular size standards; 1, crude CLC prior to enzyme digestion (20 µg); 2, CLC extract following enzyme digestion (20 µg); 3, purified CLC (20 µg). The Pro-Q Emerald stain of LPS (not shown here) looks similar to that of the Western blot, and is shown in reference 29.

Following electrophoresis the extracted CLC appeared as a large molecular size, heterogeneous smear after staining with Stains All/silver stain (though distinct bands were apparent as color initially developed) (Fig. 2B), and by Western blotting with antiserum to whole cells (Fig. 2C). Although purified LPS was clearly observed by Western blotting with antibody to O-antigen, an equivalent amount of *F. tularensis* LPS was not stained by Stains All/silver stain (probably due to the presence of only dideoxy glycoses in the O-antigen), further showing that the CLC was distinct from LPS. Furthermore, as the source of the CLC was an O-antigen negative mutant, the high molecular size material in the CLC could not be LPS. However, there was very little reactivity of the CLC with the fluorescent stain Pro-Q Emerald (Fig. 2D). The profile of the crude extract obtained following 0.5% phenol extraction showed a wide variety of proteins. However, a few low molecular size proteins were still present in the CLC following enzyme digestion, as shown by Coomassie Blue staining (Fig. 2A).

**Figure 3:**
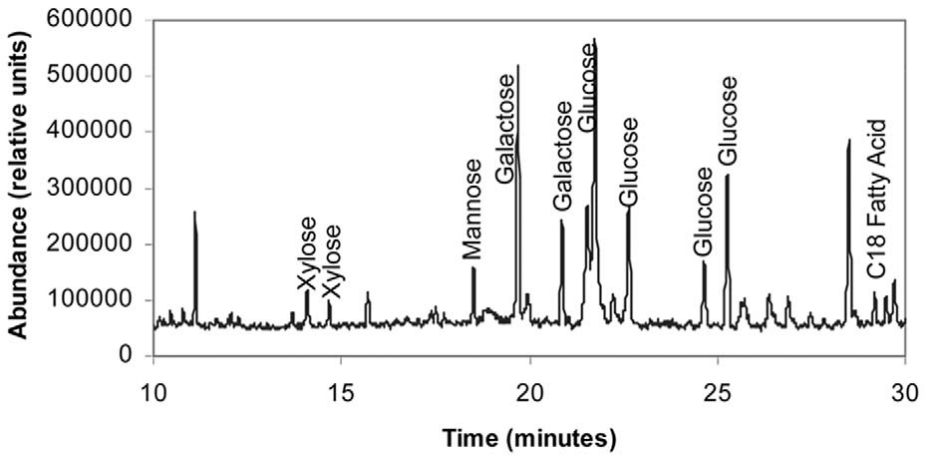
Trimethylsilyl (TMS) ethers of the methyl glycosides from purified *F. tularensis* CLC. TMS derivatives of the glycoses from the CLC were analyzed by GC/MS. All sugars and C18 fatty acids match in both retention time and mass spectrum with known standards.

The composition of the carbohydrate in the CLC was determined by GC/MS. Multiple (>10) analyses consistently indicated that the carbohydrate consisted of the neutral residues glucose, mannose, and galactose (Fig. 3). Ribose, xylose, or C18:0 fatty acids were occasional but inconsistent contaminants, and if present were removed by column chromatography. Monosaccharide residues unique to LPS, such as KDO or quinovosamine, were not present. In addition, galactose is not present in the LPS of this bacterium [20], further indicating this carbohydrate was distinct from LPS. Purified CLC was readily precipitated by addition of excess cold ethanol, further supporting that the CLC was of large molecular size. These collective results indicated that the CLC on the bacterial surface was a glycoprotein.

### Identification of the putative genes responsible for CLC carbohydrate biosynthesis

**Figure 4:**
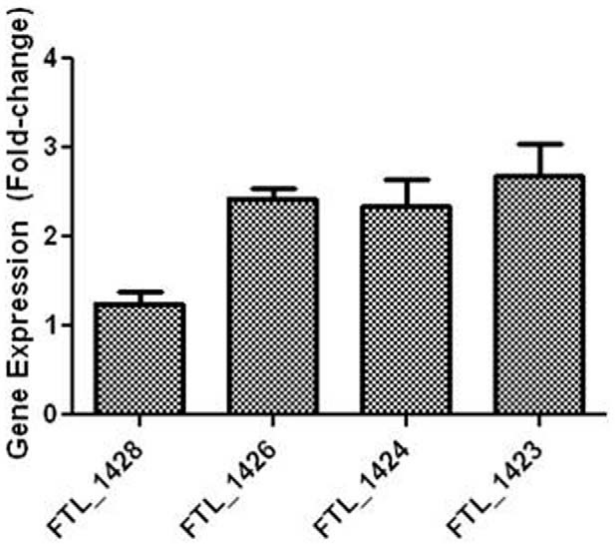
RT-qPCR of various regions of the putative CLC locus. LVS was passed in defined medium and grown on defined medium agar at 32°C in CO_2_ to maximize CLC content, or in defined medium broth at 37°C without preliminary passage to minimize CLC. The RNA was isolated, converted to cDNA, and the cDNA amplified by quantitative real-time PCR. The results are shown as the x-fold change in gene expression using LVS grown in BHIC broth to log phase (minimal CLC expression) as the calibrator and GAPDH as the endogenous control for gene expression. The locus tag of each gene from LVS is shown on the X-axis.

BLAST analysis of the *F. tularensis* LVS genome sequence identified a 12.5 kb locus containing 12 genes (FTL_1432-FTL_1421) with homology to genes that encode for proteins involved in polysaccharide synthesis (Table 1). A possible promoter was identified upstream of FTL_1432, but not anywhere else in the genetic sequence through FTL_1421. Furthermore, overlapping primers were used to “walk” down the chromosome from FTL_1432 through FTL_1421, and transcripts were obtained for every two genes (data not shown) indicating the genes in this locus are co-transcribed. Smith-Waterman analysis indicated that FTL_1423 was most similar to a galactosyl transferase from *Streptococcus pneumoniae* (34.5% amino acid identity), and FTL_1422 was most similar (35.5% identity) to a mannosyl transferase from *Salmonella enterica*. Additional evidence that this locus contributed to CLC biosynthesis was obtained by RT-qPCR (Fig. 4). The expression of FTL_1426, FTL_1424, and FTL_1423 within the putative CLC carbohydrate locus was significantly upregulated (*P* = 0.025, 0.023, and 0.031, respectively) up to three-fold when LVS was passed in CDMB and grown to maximize CLC production (cultured on Glc-CDMA at 32°C for 5 days in 7% CO_2_) compared to growth under conditions that would minimize CLC production (shaking at 200 rpm in brain heart infusion broth supplemented with 0.1% L-cysteine hydrochloride monohydrate) (BHIC) at 37°C to log phase).

**Table 1:** Putative genes and gene products that may contribute to *F. tularensis* CLC biosynthesis.

ORF in LVS	ORF in Schu S4	Size (bp)	Product^a^
FTL_1432	FTT_0789	669	D-ribulose-phosphate 3-epimerase
FTL_1431	FTT_0790	1395	Sugar transferase family protein (Glycosyl transferase)
FTL_1430	FTT_0791	1020	UDP-glucose 4-epimerase
FTL_1429	FTT_0792	1230	Glycosyl transferase group 1 family protein
FTL_1428	FTT_0793	1683	ATP-binding membrane transporter
FTL_1427	FTT_0794	1287	Hypothetical protein (Phosphoserine phosphatase)
FTL_1426	FTT_0795	684	Hypothetical protein (protein cfa)
FTL_1425	FTT_0796	762	Hypothetical protein
FTL_1424	FTT_0797	960	Glycosyl transferase family protein (galactosyl transferase)
FTL_1423^b^	FTT_0798	1008	Glycosyl transferase family protein (galactosyl transferase)
FTL_1422^b^	FTT_0799	1014	Glycosyl transferase family protein (mannosyl transferase)
FTL-1421	FTT_0800	663	Haloacid dehalogenase

a Determined by Smith-Waterman analysis

b Deletion of both of these genes resulted in CLC-deficient mutant LVSΔ1423/1422.

### Mutagenesis of FTL_1423/1422

**Figure 5:**
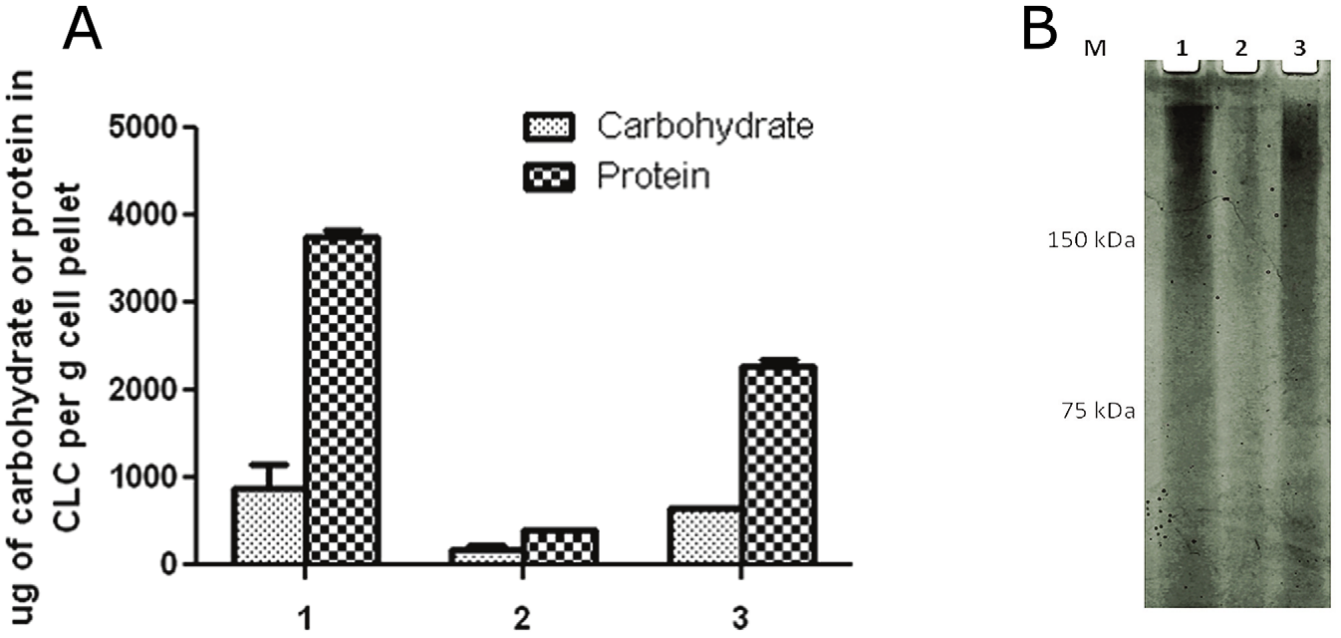
CLC content from LVS_P10, LVSΔ1423/1422_P10, and LVSΔ1423/1422[1423/1422^+^]_P10. Lanes: 1, LVS_P10; 2, LVSΔ1423/1422_P10; 3, LVSΔ1423/1422[1423/1422^+^]_P10. A) Carbohydrate and protein content of extracted CLC; B) Stains All/silver stain of extracted CLC. The reducing carbohydrate content was measured by phenol sulfuric acid assay [47], and the protein content was measured by BCA assay. The CLC was extracted from the same number of cells of each strain, as described in Materials and Methods.

FTL_1423 and FTL_1422 were deleted in LVS and WbtI_G191V__P17 by allelic exchange (see Materials and Methods). Mutagenesis was confirmed by the inability to amplify either open reading frame (ORF) by polymerase chain reaction (PCR), by identification of the kanamycin resistance gene from the suicide vector in the genome (by PCR and colony blot hybridization), and by sequencing of PCR products (data not shown). Unlike LVS_P10 (Fig. 1A and 1E) and WbtI_G191V__P17 (Fig. 1G), LVSΔ1423/1422_P10 and WbtI_G191V__P17Δ1423/1422 lacked any evidence of a CLC following passage to enhance CLC synthesis (Fig. 1C and 1H, respectively). Furthermore, significantly less CLC carbohydrate (*P* = 0.01) and protein (*P*<0.01) was extracted from LVSΔ1423/1422_P10 with 0.5% phenol than the parent (Fig. 5A), and 91% less glucose, galactose, and mannose were present in extracts, as determined by GC/MS (data not shown). Gel electrophoresis of the CLC extracts from LVS_P10 and LVSΔ1423/1422_P10 confirmed the mutant was CLC-deficient (Fig. 5B). To confirm that deletion of FTL_1423/1422 was solely responsible for the absence of the CLC, both ORFs were cloned into expression vector pFNLTP6 [39], which were then introduced into LVSΔ1423/1422 by electroporation. The complemented mutant (LVSΔ1423/1422[1423/1422^+^]_P10] (passed 10 times in CDMB) was restored in CLC synthesis, as shown by electron microscopy (Fig. 1D), by enhancement of the protein (partially) and carbohydrate content of purified CLC (Fig. 5A), and by Stains All/silver stain following gel electrophoresis (Fig. 5B).

**Figure 6:**
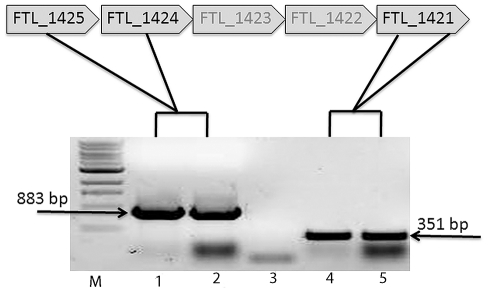
RT-PCR of the DNA region FTL_1421 from LVSΔ1423/1422. LVS and LVSΔ1423/1422 were grown on Glc-CDMA for at least 2 days, the RNA isolated, converted to cDNA, and amplified by PCR to determine if a transcript from DNA downstream of the mutation was made. Lanes: M, 1 kb+ DNA molecular size standards; 1, control amplification of FTL_1425-1424 in LVS; 2, amplification of FTL_1425-1424 upstream of the mutation in LVSΔ1423-1422; 3, control amplification of FTL_1425-1424 upstream of the mutation (no bacteria); 4, control amplification of FTL_1421 from LVS; 5, amplification of FTL_1421 immediately downstream of the mutation in LVSΔ1423/1422. The presence of a normal band of about 351 bp from LVSΔ1423/1422 indicated that the mutation had no polar effect on downstream genes.

To confirm that the deletion of FTL_1423 and FTL_1422 did not have a polar effect on downstream genes, a DNA region from FTL_1421 from the parent and the mutant was subjected to RT-PCR (Fig. 6). The transcript of this region was identical to that of the transcript from LVS, indicating that genes downstream of FTL_1423 and FTL_1422 were transcribed and not responsible for the loss of CLC in mutant LVSΔ1423/1422. A separate control containing *taq* polymerase and all other reagents except the reverse transcriptase confirmed that the band was not genomic DNA (not shown).

### *In vitro* growth rate and serum resistance of LVSΔ1423/1422

**Figure 7:**
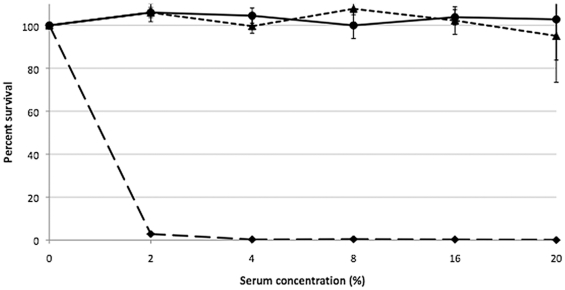
Bactericidal assay of LVSΔ1423/1422 and control strains in the presence of fresh human serum. The bacteria were diluted in PBS supplemented with 0.15 mM CaCl_2_, 0.5 mM MgCl_2_, and 2%, 4%, 8%, 16%, or 20% fresh, pooled human serum. Aliquots were cultured by viable plate count before and after 60 min. incubation at 37°C. Bacterial strains: LVS, **^_____^•^_____^**; LVSΔ1423/1422, ---▴---; O-antigen mutant WbtI_G191V__P17, ^__ __^ **♦^__ __^**.

The generation time for strain LVS in BHIC was approximately 2.5 h during log phase. However, mutant LVSΔ1423/1422 grew substantially slower, with a generation time of approximately 6 h. Both parent LVS and mutant LVSΔ1423/1422 were completely resistant to the bactericidal action of up to 20% fresh guinea pig serum and human serum (v/v), whereas there was 0% survival of passed LVS O-antigen mutant WbtI_G191V__P17 [11] in as little as 2% human serum (Fig. 7). Therefore, the CLC did not contribute to serum resistance.

### Viability of LVS, LVSΔ1423/1422, and LVSΔ1423/1422[1423/1422^+^] in macrophages

**Figure 8:**
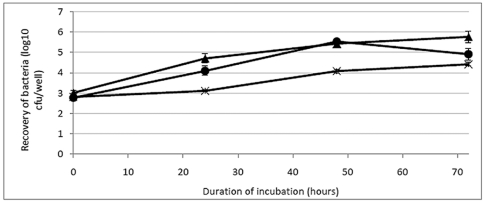
Intracellular survival of *F. tularensis* LVSΔ1423/1422 in J774A.1 cells. The J774A.1 monolayer (approximately 4.5×10^5^ macrophages/well) was infected with approximately 5.5×10^7^ CFU/well of strain LVS (**•**), LVSΔ1423/1422 (▴), or LVSΔ1423/1422[1423/1422^+^] (**X**). Intracellular survival of the bacteria was determined at 0, 24, 48, and 72 h post-infection, as described in Materials and Methods. Data are shown on the log scale as the average number of bacteria recovered from dilutions of lysates of J774A.1 cells. The results shown were from two experiments tested in duplicate at each time point. The slopes for intracellular growth of the bacteria as log_10_ CFU/well between the 24^th^ and the 48^th^ hour for strains LVS, LVSΔ1423/1422, and LVSΔ1423/1422[1423/1422^+^] were +1.44, +0.73, and +0.97, respectively.

By 24 hours after inoculation, there was no obvious difference in viability between LVS and LVSΔ1423/1422, but growth of complemented strain LVSΔ1423/1422[1423/1422^+^] was delayed in J774A.1 cells assayed on separate days. However, the slopes for intracellular growth of the bacteria as log_10_ CFU/well between 24 and 48 h for strains LVS, LVSΔ1423/1422, and LVSΔ1423/1422[1423/1422^+^] were similar (Fig. 8). Thus, the CLC did not appear to be required for growth in murine macrophages.

### Virulence of LVSΔ1423/1422 in mice

**Figure 9:**
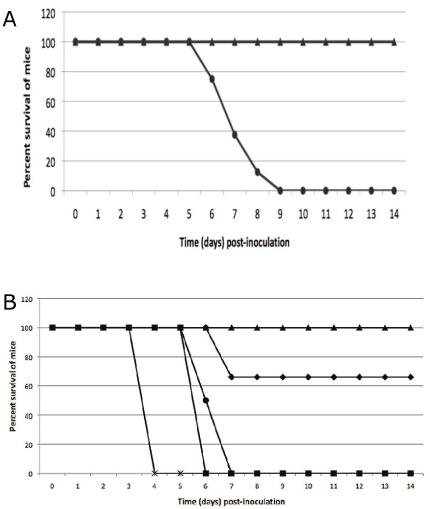
Survival of mice inoculated with *F. tularensis* LVSΔ1423/1422. Groups of BALB/c mice were inoculated IN (A) or IP (B), and survival was monitored for six weeks. No mice challenged IN with LVSΔ1423/1422 died during the study. The doses and symbols used for IN inoculations were about 1.2×10^4^ CFU/mouse of LVS (**•**), and about 1.6×10^4^ CFU of LVSΔ1423/1422 (▴). The doses and symbols used for IP inoculations were 262 CFU/mouse of LVS (**•**); 3,375 CFU/mouse of LVS (x); 1,124 CFU/mouse of LVSΔ1423/1422 (▴); 11,136 CFU/mouse of LVSΔ1423/1422 (**♦**); 33,408 CFU/mouse of LVSΔ1423/1422 (▪).

BALB/c mice were inoculated by the intranasal (IN) or IP routes with LVS or LVSΔ1423/1422 to evaluate the effect of loss of CLC on *F. tularensis* virulence (Fig. 9). All mice inoculated IN with about 1.2×10^4^ CFU of LVS died or needed to be euthanized in less than 10 days. In contrast, all mice inoculated IN with up to 1.6×10^4^ CFU of LVSΔ1423/1422 survived longer than six weeks and never developed clinical symptoms (Fig. 9A).

All BALB/c mice inoculated IP with 41, 262 or 3,375 CFU of LVS died within 7 days. In contrast, all mice inoculated IP with 1100 CFU or 2 of three mice inoculated IP with 11,136 CFU of strain LVSΔ1423/1422 survived longer than four weeks. However, all mice inoculated IP with 33,408 CFU of the mutant died or were euthanized within 6 days (Fig. 9B). Although the lethal dose of the mutant was lower following IP challenge than IN challenge, the LD_50_ of the parent was also much lower following IP challenge (<41 CFU) than IN challenge (∼200 CFU).

### Persistence of LVSΔ1423/1422 in mouse tissues

**Figure 10:**
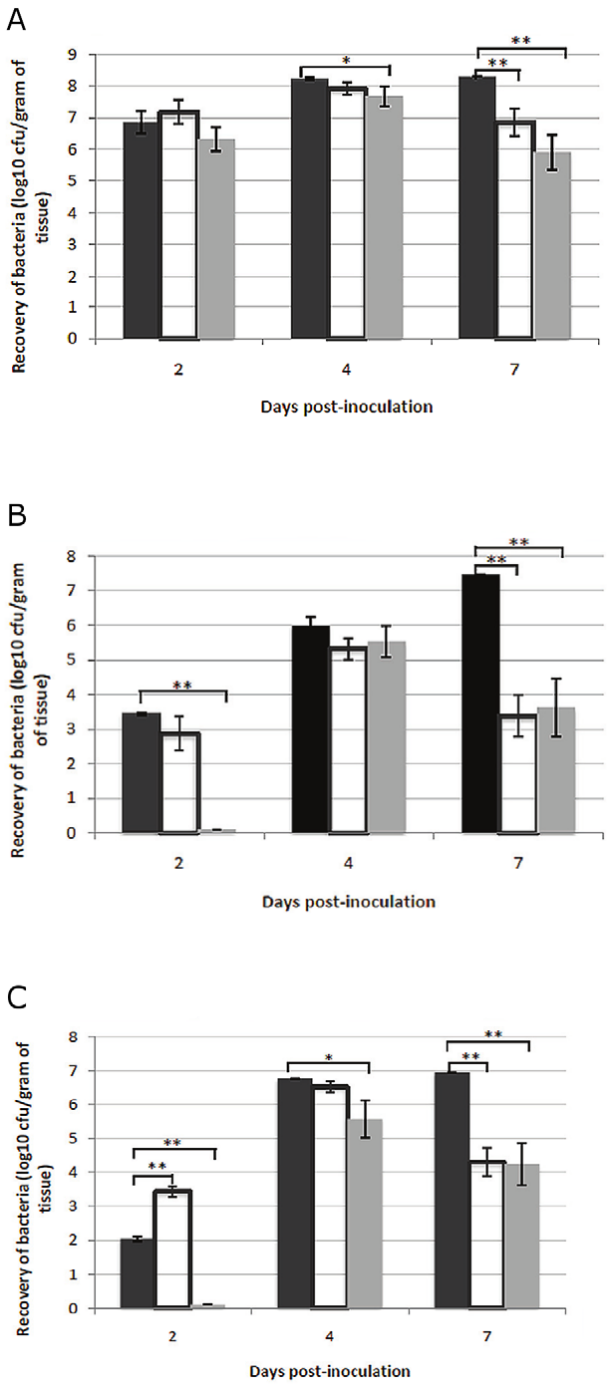
Recovery of *F. tularensis* LVSΔ1423/1422 from the tissues of mice following IN inoculation. Groups of BALB/c mice were inoculated IN with 7.9×10^3^ CFU of strain LVS, 5.0×10^4^ CFU of strain LVSΔ1423/1422 (high dose of mutant), or 1.1×10^4^ CFU of strain LVSΔ1423/1422 (low dose of mutant). At 2, 4, or 7 days PI, mice were euthanized. The lungs (A), liver (B), and spleen (C) were aseptically removed, homogenized in PBS, and the CFU/g of tissue determined. The recovery of bacteria from inoculated mice are shown as dark-filled bars (LVS), open bars (high dose of LVSΔ1423/1422), and grey-filled bars (low dose of LVSΔ1423/1422). The mean values of the CFUs of each dose of the mutant were separately compared with LVS. The *P* values for the differences between the mean values were <0.05 (*) or <0.005 (*).

BALB/c mice were inoculated IN with 7.9×10^3^ CFU of LVS, 5.0×10^4^ CFU of LVSΔ1423/1422 (high dose), or 1.1×10^4^ CFU of LVSΔ1423/1422 (low dose). The mice were euthanized at 2, 4, or 7 days post-inoculation (PI), and the number of bacteria in the lungs, liver, and spleen determined (Fig. 10). LVS numbers increased in all three organs between day-2 and day-7 PI. LVSΔ1423/1422 from the high dose challenge was recovered from lungs (Fig. 10A) and liver (Fig. 10B) in approximately the same numbers as LVS on day-2 and day-4 PI, but was recovered in significantly fewer numbers from lungs, liver, and spleen on day-7 PI (*P*<0.005 for each organ). Of interest was that from the high dose challenge 1.5 logs more of the mutant than the parent was recovered from the spleen at day 2 PI (Fig. 10C). This difference may have been due to the dose of the mutant being 5 times higher than that of the parent, and the spleen concentrating bacteria entering the blood stream. However, recovery of LVSΔ1423/1422 from the spleen after low dose challenge (similar to the LVS challenge dose) on day 2 PI was significantly lower. While similar numbers of the mutant from low dose challenge were present in the lungs at day 2 PI, recovery dropped off to significantly fewer numbers by day 4 PI (*P*<0.05), and to a highly significant difference by day 7 PI (*P*<0.005) (Fig. 10A). However, very few mutant bacteria from the low dose challenge were recovered from the liver or spleen by day 2 PI, were present in similar numbers as the parent in the liver by day 4 PI (significantly fewer numbers in the spleen; *P*<0.05), and fewer numbers in the liver and spleen (highly significant) by day 7 PI (*P*<0.005) (Fig. 10B and 10C, respectively). These results indicated that following IN challenge it took longer for similar numbers of the CLC mutant (compared to the parent) to multiply and migrate from the lungs to internal organs, could persist there for a few days, but unlike the parent, began to be eliminated from the host. Thus, the expression of the CLC was essential for full virulence of LVS in the respiratory tract.

In a separate trial, C57BL/6 mice were inoculated with a mixture of 5.9×10^3^ CFU/mouse of strain LVS mixed with 8.0×10^3^ CFU/mouse of mutant LVSΔ1423/1422. Five days PI the mice were euthanized, tissue homogenates were cultured on BHIC agar supplemented with 5% (v/v) sheep blood (BHICB), and an average of 1.0×10^5^, 1.9×10^7^, and 1.3×10^7^ of bacteria were present per gram of liver, lungs, and spleen, respectively (Table 2). The same tissue extracts plated on BHICB plates containing kanamycin yielded 0, 0, and 6.1×10^2^ colonies per gram of liver, lung, and spleen, respectively (Table 2). Therefore, LVSΔ1423/1422 was significantly less fit (*P*<0.005 from each organ) to survive in host tissues than LVS.

**Table 2:** Recovery of LVS and LVSΔ1423/1422 following co-inoculation into C57BL/6 mice^a^.

Organ	CFU on BHICB	CFU on BHICB containing Kanamycin
Liver	1.0×10^5^	0.00
Lungs	1.9×10^7^	0.00^b^
Spleen	1.3×10^7^	6.1×10^2^

a Five mice were inoculated IN with a mixture of 5.9×10^3^ CFU of LVS and 8.0×10^3^ CFU of LVSΔ1423/1422. Five days PI the mice were euthanized, and tissue extracts were cultured on BHICB with or without Kan. The numbers shown represent the average CFU/g tissue.

b A few colonies were isolated from the lungs of one of five mice.

### Protective efficacy of LVSΔ1423/1422 in mice

**Figure 11:**
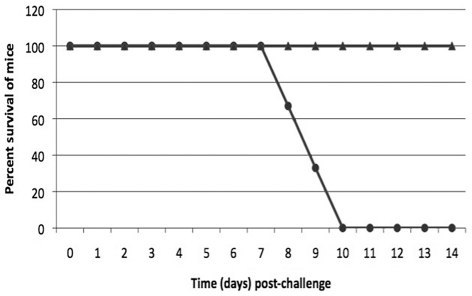
Protective efficacy of LVSΔ1423/1422 against IN challenge of mice with LVS. Groups of BALB/c mice were injected with PBS (•), or inoculated with 6.1×10^3^ CFU/mouse of LVS Δ1423/1422 (**▴**). Six weeks PI, the mice were challenged IN with 1.4×10^5^ CFU/mouse of LVS, and the mice were monitored for 4 weeks. No mice died or were symptomatic by 10 days post-challenge. The *P* value for animal survival after 10 days post-challenge was <0.001.

Mice inoculated once IN with 6.1×10^3^ CFU of LVSΔ1423/1422 were challenged with 1.4×10^5^ CFU of LVS IN six weeks PI. All mice survived challenge (Fig. 11) and none developed clinical symptoms, whereas all control mice died or had to be euthanized within 10 days. Thus, LVSΔ1423/1422 lacking CLC was capable of inducing significant protection in BALB/c mice following IN challenge with a high dose of LVS by 10 days post-challenge (*P*<0.001). Mice previously inoculated IP with 39 CFU of LVSΔ1423/1422 were challenged IN 7 weeks PI with 7.9×10^3^ CFU of LVS. The challenged mice developed mild clinical symptoms (reduced activity) until about six days PI, after which time they recovered (data not shown). Therefore, IP inoculation with a low dose of 39 CFU/mouse also induced protection against respiratory tularemia.

## Discussion

*F. tularensis* has long been postulated to be encapsulated, based primarily on electron microscopy [20], [21], [27]. However, the electron dense material around *F. tularensis* is not always evident and has not been isolated, raising question as to whether a true capsule actually exists. Cherwonogronzky *et al*. [27] showed that a CLC can be enhanced by daily passage of *F. tularensis* LVS in CDMB, followed by culture on CDMA, and that bacteria enhanced for this surface material are more virulent in mice. We confirmed by EM and by RT-qPCR that the CLC was upregulated when LVS was passed in CDMB and grown at lower temperature in CO_2_ for several days on CDMA, compared with when the bacteria were grown shaking rapidly in complex broth medium. A similar CLC could also be isolated and observed by EM around type A strains SCHU S4 and TI0902. Three of four ORFs within the putative CLC locus (FTL_1423/1424/1426) were upregulated more than 2 to 3-fold when the bacteria were grown to enhance CLC synthesis, but one ORF (FTL_1428) was not. In data not shown FTL_1428 was also deleted by allelic exchange, but no significant difference in phenotype or virulence in mice could be identified between LVSΔ1428 and LVS. Smith-Waterman analysis indicated that FTL_1428 has homology to a family of ATP-binding cassette (ABC) transmembrane transporters. Our hypothesis was that deletion of FTL_1428 may abolish export of the CLC to the surface without inhibiting synthesis. As this was not the case it is possible that FTL_1428 is not functional or does not function in CLC export or regulation. Therefore, it was not surprising that FTL_1428 was not upregulated during enhanced CLC expression.

Analysis by GC/MS, chemical assays, amino acid analysis, and electrophoretic analysis using the carbohydrate-specific fluorescent stain Pro-Q Emerald indicated that unlike most bacterial capsules, the predominant component of the CLC was protein, not carbohydrate. Furthermore, following extraction with 0.5% phenol, the CLC foamed and easily became insoluble. These are also features of self-assembling bacterial surface (S)-layer proteins, which are common in bacteria and are often glycosylated [40]. Proteinase K digestion was used to remove most of the protein to improve solubility and focus on the carbohydrate component, though some proteins were proteinase K resistant. Amino acid analysis indicated that the majority of the amino acids remaining after Proteinase K digestion were glutamic and aspartic acids (unpublished data). Supplementation of the growth medium with glucose further enhanced CLC synthesis, which is consistent with the effect of glucose supplementation on cell surface carbohydrate content [41]. Repeated analyses of the carbohydrate component of the CLC consistently yielded glucose, galactose, and mannose. Although the structure of the carbohydrate polymer has not yet been determined, gel electrophoresis and column chromatography confirmed that the material is of large molecular size. Bacterial glycoproteins may have only 150 glycoses, but are attached as heterogeneous repeating polymers, resulting in a ladder-like banding pattern or smear in polyacrylamide gels [40]. A similar ladder-like/smear profile was observed with the *F. tularensis* CLC following staining with Stains All/silver stain and Western blotting. Therefore, glucose, galactose, and mannose might compose a trisaccharide polymer of a glycosylated protein. Balonova *et al.* [42] have confirmed that glycosylated proteins are present in *F. tularensis*. PilA and at least 14 additional proteins were determined to be glycosylated by at least two methods. Although the CLC may not have been upregulated in the bacteria used in their studies, it is apparent that glycoproteins are common in *F. tularensis*.

The CLC was distinct from the LPS, as determined by GC/MS, lack of more LPS on cells enhanced for CLC, and the molecular size of CLC isolated from an O-antigen mutant. Apicella *et al*. [29] recently reported the presence of an O-antigen capsule on *F. tularensis*, which was detected by monoclonal antibody (MAb) binding. This MAb (11B7) bound to the crude CLC initially extracted from the surface of LVS_P10, but not from O-antigen mutant WbtI_G191V__P17, or from the purified CLC (unpublished data). Therefore, the O-antigen capsule is also distinct from this CLC. “Unencapsulated” mutants of *F. tularensis* LVS have been described that are highly susceptible to complement-mediated killing [21], [23]. However, these mutants may also have been O-antigen deficient because *F. tularensis* LVS lacking O-antigen and/or O-antigen capsule is highly serum-sensitive [10], [11], [12], [13], [30]. In contrast, the CLC mutant generated in this study was resistant to the bactericidal action of guinea pig or human serum.

Loci that are responsible for the synthesis and export of bacterial carbohydrate polymers are about 12–18 kb in size, and contain genes that encode for ABC transporters, glycosyltransferases, membrane spanning proteins, etc. [43], [44]. Larsson *et al*. [32] described a putative polysaccharide locus from the genome sequence of *F. tularensis* strain SCHU S4. To determine if this locus contributed to CLC biosynthesis in LVS, FTL_1423 and FTL_1422 (which have homology to genes encoding for glycosyl transferases) were mutated by allelic exchange. The deletion of these genes appeared adequate to block assembly of the CLC on the bacterial surface, as no CLC could be observed by EM around LVSΔ1423/1422_P10, and little CLC could be isolated from this mutant. Therefore, glycosylation of protein may be required for formation of the CLC on the surface. Complementation of LVSΔ1423/1422 *in trans* with both genes restored CLC expression, and RT-PCR indicated that expression of the gene downstream of the mutation was not affected, confirming that loss of the CLC was not due to another mutation.

The capability of mutant LVSΔ1423/1422 to survive and grow in the mouse macrophage-like cell line J774A.1 for 72 h PI was similar to that of parent strain LVS. However, growth of the complemented mutant in macrophages was delayed during the first 24 h, after which time LVSΔ1423/1422[1423/1422^+^] grew at a similar rate as the parent and mutant. This growth lag may have been due to catabolic effects and additional energy needed to synthesize proteins by genes expressed on the plasmid *in trans*. Nonetheless, the loss of CLC did not appear to substantially interfere with intra-macrophage growth, in contrast to mutants that fail to make LPS O-antigen or O-antigen capsule [30], supporting the distinction between these surface structures.

Deletion of both FTL_1423 and FTL_1422 in LVS resulted in significant loss of virulence in mice following IN challenge. These results are consistent with those of Weiss *et al*. [45], who reported that transposon mutagenesis of *F. novicida* FTN_1213 (equivalent to FTL_1423 of LVS) resulted in moderate attenuation following subcutaneous challenge of mice. The CLC mutant was also highly attenuated following IP challenge. Inoculation with 11,136 CFU of the mutant was required to cause skin ruffling and death of some mice, which is a lower dose than that required for clinical symptoms by the IN route. However, parent strain LVS is also much more virulent for mice by the IP route than the intravenous or subcutaneous routes [46]. Inoculation by the IP route introduces the bacteria directly into the systemic tissues and bypasses many of the innate immune defenses.

At 2 days and 4 days post-IN inoculation, the presence of LVSΔ1423/1422 in the lungs, liver, and spleen was not highly significantly different from that of LVS at high or low dose challenge. However, the numbers of this mutant in all three organs dropped significantly by 7 days PI, even after high dose inoculation. However, it took significantly longer for bacteria from the low dose challenge to migrate to the liver and spleen from the lungs before growing to similar numbers as the parent, and then began to be cleared. Thus, the CLC was necessary for *F. tularensis* LVS to persist in the tissues. Furthermore, when LVS and LVSΔ1423/1422 were inoculated concurrently into mice, the mutant was unable to compete with the parent and few mutant cells could be recovered from mouse tissues.

After a single IN inoculation with a high dose of LVSΔ1423/1422 (up to 80× the LVS LD_50_), mice challenged with LVS IN with >700 times the LD_50_ developed no clinical symptoms of tularemia. In addition, mice inoculated IP with LVSΔ1423/1422 were also protected against low dose IN challenge with LVS, demonstrating that systemic immunity to this mutant was adequate for protection in the respiratory tract. Therefore, the generation of CLC mutants in type A strains is warranted to determine if such mutants are adequately attenuated and capable of inducing a protective immune response against type A strains.

These results showed that the CLC may be a glycoprotein that is upregulated under particular growth conditions, the glycose component of the CLC contained glucose, galactose, and mannose, the loci identified as FTL_1432 through FTL_1421 in LVS contribute to CLC synthesis, and that the CLC is required for full virulence of LVS, but not for inducing protective immunity in mice against LVS.

## Materials and Methods

### Ethics statement

All proposals involving the use of living vertebrates are reviewed by the Virginia Tech Institutional Animal Care and Use Committee to assure humane care and treatment of the animals involved. Approved proposals comply with "U.S. Government Principles for the Utilization and Care of Vertebrate Animals Used in Testing, Research, and Training, The Animal Welfare Act, As Amended, The Public Health Service (PHS) Policy on Humane Care and Use of Laboratory Animals, "Virginia Tech Policies Governing the Use of Animals in Research and Teaching". Virginia Tech has a written, approved Animal Welfare Assurance on file with the PHS Office of Laboratory Animal Welfare (OLAW). The university's Animal Welfare Assurance number is A-3208-01, expiration date 3-31-2012. All experiments with animals were approved by the Virginia Tech Institutional Animal Care and Use Committee under approved protocol 08-257-CVM.

### Bacterial strains and growth conditions

The bacterial strains used and their sources are listed in Table S1. *Escherichia coli* DH5α was grown in Luria–Bertani (LB) medium (Becton-Dickinson, Franklin Lakes, NJ) at 37°C containing 100 µg ampicillin (Amp)/ml or 50 µg kanamycin (Kan)/ml for selection of recombinant strains. *F. tularensis* strains were cultured from frozen stock suspensions onto BHIC agar (Becton-Dickinson and Sigma-Aldrich, St. Louis, MO) or BHICB, and incubated at 37°C in 7% CO_2_, unless otherwise stated. For culture in broth, *F. tularensis* strains were grown with shaking (175 rpm) in BHIC broth at 37°C, or Glc-CDMB [27] at 32°C. For CLC preparation *F. tularensis* LVS_P10 or WbtI_G191V__P17 was grown on Glc-CDMA in petri dishes (150 mm×15 mm), and incubated at 32°C in 7% CO_2_ for 5 days. All experiments with LVS and mutants were carried out in biosafety level (BSL)-2 facilities in an approved biosafety cabinet.

### Extraction of LPS

LPS was purified from *F. tularensis* LVS by aqueous phenol extraction, enzyme digestion, and ultracentrifugation from killed cells, as described previously [11].

### Purification of CLC

The CLC was extracted from O-antigen LVS mutant WbtI_G191V_ that was passed daily 17 times in CDMB (WbtI_G191v__P17) to avoid contamination with LPS O-antigen. The cells were grown in Glc-CDMB to mid-log phase, 500 µl was streaked onto large petri plates of Glc-CDMA, and the plates were incubated for 5 days at 32°C in 7% CO_2_. Approximately 10 g of bacterial cells (wet weight) were gently resuspended into 200 ml of 0.5% phenol (in water) and incubated at room temperature for 10 min. A thick, foamy extract was obtained and subjected to centrifugation at 10,000×*g* for 15 min to remove cells, followed by centrifugation of the supernatant again. The crude CLC was precipitated by addition of 60 mM sodium acetate and 5 volumes of cold (−20°C) ethanol, and incubation at −20°C overnight. The precipitate was sedimented by centrifugation at 10,000×g for 15 min and the pellet suspended in 50 ml of 50 mM Trizma base (pH 7.3) containing 10 mM CaCl_2_, 10 mM MgCl_2_, and 0.05% sodium azide. Ten microliters of RiboShredder RNase (Epicentre, Madison, WI) was added, and the mixture incubated for 2 hours at 37°C. Twenty-five µg/ml of DNase (Sigma-Aldrich, St. Louis, MO) was added and the incubation continued for 1 hour. One hundred µg/ml of Proteinase K (Sigma-Aldrich) was added and the mixture incubated for 2 hours at 37°C, followed by incubation at 55°C overnight. Impurities were removed from the semi-purified complex by ultracentrifugation at 100,000×*g* at 4°C for five hours to overnight. The supernatant was dialyzed in a 50-kDa membrane against four changes of distilled water and lyophilized. In some cases the CLC was further purified by S-300 column chromatography with water or 0.1% sodium dodecyl sulfate as eluent (15 mm×252 mm; GE Healthcare Life Sciences, Piscataway, NJ), and the carbohydrate-positive fractions were dialyzed and lyophilized.

### CLC compositional analysis

CLC samples were extracted from the same number of cells, as determined by optical density and viable plate count, and analyzed by phenol-sulfuric acid assay [47] for carbohydrate content, KDO assay [48] and Western blotting [11] for LPS, bicinchoninic acid assay (BCA) for protein content (Pierce), and galactose oxidase assay for galactose (Invitrogen). Glycosyl composition was determined by combined GC/MS of the per-O-trimethylsilyl (TMS) derivatives of the monosaccharide methyl glycosides produced from the sample by acidic methanolysis, as described [49].

### Electrophoretic analysis and Western blotting

The electrophoretic profile of the CLC was resolved by sodium dodecyl sulfate-polyacrylamide gel electrophoresis using Novex® 4–12% Pre-Cast bis-Tris gels (Invitrogen, Carlsbad, CA). Following electrophoresis, the gel was fixed in 25% isopropanol/10% acetic acid overnight and stained with 0.25% Stains All (Sigma-Aldrich, St. Louis, MO) for 2 hours [50]. The bands were visualized on a lightbox, color and size noted, and the gel silver stained as described [50]. Separate gels were stained with Coomassie Blue (Pierce, Rockford, IL), or the samples were transferred to nitrocellulose using an X-Cell II Blot Module Semi-Dry Transfer unit (Invitrogen) for Western blotting. Blots were developed using rabbit polyclonal antiserum to LVS (1∶10,400 dilution) [51], followed by anti-rabbit IgG coupled to horseradish peroxidase (HRP; Jackson ImmunoResearch Labs) (1∶2,000 dilution), and developed with 3,3,5,5-tetramethylbenzidine (TMB; Pierce). The presence of carbohydrate in the gel was also examined by staining with Pro-Q Emerald 300 (Molecular Probes, Eugene, OR).

### Negative stain electron microscopy

All *F. tularensis* strains were passed in CDMB to enhance CLC as described for WbtI_G191V__P17. *F. tularensis* strains were grown on Glc-CDMA for 5 days at 32°C. The cells were gently scraped into sodium cacodylate buffer containing 3% glutaraldehyde and turned end-over-end for 2 hours. The cells were washed, suspended in 10 mM sodium cacodylate buffer, adhered to formvar-coated grids, stained with 0.5% uranyl acetate, and viewed with a JEOL 100 CX-II transmission electron microscope [52].

### DNA sequence analyses

Annotation of putative *F. tularensis* LVS genes was determined using BLAST [53] and the Smith-Waterman algorithm [54].

### DNA manipulation

DNA extraction and manipulation procedures were carried out as described [55]. Restriction enzymes and T4 DNA ligase were obtained from New England BioLabs (Ipswich, MA). Plasmid DNA was extracted using the QIAprep^®^ Spin Miniprep and QIAquick^®^ Gel, as described by the manufacturer (QIAGEN, Valencia, CA). Genomic DNA from *F. tularensis* LVS was purified using the PUREGENE™ DNA Isolation Kit (Gentra Systems, Minneapolis, MN). The StrataClone™ PCR cloning kit (Stratagene™, La Jolla, CA) was used for PCR cloning. Oligonucleotides were obtained from Integrated DNA Technologies, Inc., Coralville, IA.

### Construction of *F. tularensis* LVS allelic exchange mutants

Open reading frames FTL_1423 and FTL_1422 of strains LVS and WbtI_G191V__P17 were deleted by allelic exchange. A 1.3-kb region upstream of FTL_1423 was amplified by PCR using the primer pair (containing restriction enzyme sites) FTL1424_F_SalI and FTL1423_R_StuI. A similar size region downstream of FTL_1422 was separately amplified by PCR using the primer pair FTL1422_F_StuI and FTL1421_R (Table S2). The two PCR products were ligated to each other by fusion PCR using Taq polymerase, and then cloned into TA cloning vector pSC-A (Stratagene) to produce pSC-1423/1422. This plasmid was isolated from *E. coli* DH5α grown on LB agar containing 100 µg/ml of Amp. The Tn*903 npt* gene [56] that confers Kan resistance (Kan^r^) was isolated from pUC4K by digestion with *Pvu*II, and cloned into *Stu*I-digested (and blunt ended) plasmid pSC-1423/1422, which resulted in the Kan^r^ gene being inserted between FTL_1423 and FTL_1422. The resulting plasmid was isolated from *E. coli* DH5α grown on LB agar containing 100 µg/ml Kan, designated pSC-1423/1422K, and transformed into *F. tularensis* LVS or WbtI_G191V__P17 by cryotransformation [57]. Colonies were collected from the plates and subcultured on BHICB containing 8 µg/ml of Kan for 6 days at 37°C in 5% CO_2_. Deletion of FTL_1423 and FTL_1422 and the presence of the Kan^r^ gene from selected Kan^r^ colonies were determined by PCR. Confirmation of the deletion was done by sequencing of the region from FTL_1424 to FTL_1421 at the Virginia Bioinformatics Institute core sequencing facility at Virginia Tech. One verified recombinant of each strain was selected and designated LVSΔ1423/1422 (derived from LVS) and WbtI_G191V__P17Δ1423/1422 (derived from O-antigen mutant WbtI_G191V__P17). The *capB* gene from these mutants was amplified by PCR, confirming they were *F. tularensis* (data not shown).

For complementation, the entire FTL_1423-FTL_1422 region was amplified by PCR and cloned into expression vector pFNLTP6 [39] to produce pFTAB-1. FTL_1423/1422 was transcribed under the *groE* promoter of pFNLTP6. The *cat* gene encoding resistance to chloramphenicol and transcribed under control of its native promoter was amplified by PCR from plasmid pBBR1MCS [58] and cloned into the *Pst*I site of pFTAB-1 to produce pFTAB-2. This plasmid was introduced into LVSΔ1423/1422 by cryotransformation [57]. Colonies resistant to 10 µg/ml of chloramphenicol were subcultured, and a clone containing the recombinant plasmid (determined by restriction enzyme digestion) was designated LVSΔ1423/1422[1423/1422^+^].

### Reverse-transcriptase polymerase chain reaction (RT-PCR) and reverse-transcriptase quantitative PCR (RT-qPCR)

RNA was isolated from *F. tularensis* LVS strains using the RNeasy extraction kit following digestion of cells with 400 µg/ml of lysozyme, as described (Qiagen, Valencia, CA). RNA integrity values of 9.2–9.8 were obtained using the BioAnalyzer (Agilent, Santa Clara, CA). For RT-PCR, bacteria were grown on Glc-CDMA at 37°C for at least 2 days prior to extraction of RNA. RNA was converted to cDNA and amplified with the SuperScript III First Strand Synthesis System (Invitrogen, Carlsbad, CA) using the corresponding primers listed in Table S3.

For RT-qPCR, bacteria were cultured on Glc-CDMA at 32°C in 7% CO_2_ for 5 days or cultured in BHIC broth at 37°C with shaking overnight to enhance or minimize CLC production, respectively. RNA was converted to cDNA using the random priming method of the High Capacity cDNA Reverse Transcription Kit (Applied Biosystems-AB, Foster City, CA). Real-time PCR reactions were performed in 25 µl reactions using Power Sybr Green (Applied Biosystems) and gene-specific primers (Table S3). RT-qPCR reactions were carried out in a 7300 Real-Time PCR System and analyzed using the SDS Software package (Applied Biosystems) using BHIC broth-grown LVS shaking at 37°C as the calibrator and GAPDH as the endogenous control for gene expression. Biological and technical replicates were done in triplicate.

### Serum bactericidal assay

The bactericidal activity of 20% fresh guinea pig serum or various dilutions of human serum for *F. tularensis* mutants was determined as previously described [59]. Controls included LVS and LVS O-antigen mutant WbtI_G191V_ [11], exposed to 20% fresh serum or 20% heat-inactivated serum.

### **Survival of** ***F. tularensis*** **LVS and mutants in macrophages**

The intracellular survival and growth of LVS, LVSΔ1423/1422, and LVSΔ1423/1422[1423/1422^+^] was determined in the murine macrophage-like cell line J774A.1 (American Type Culture Collection, Manassas, VA) by modification of published methods [60]. The bacteria were mixed with macrophages at a multiplicity of infection of 100∶1 (bacteria:macrophages). After 1 h incubation at 37°C in 5% CO_2_, extracellular bacteria were removed by washing the cells with PBS, and the medium was replaced with 1 ml of complete Dulbeccos's Modified Eagle Medium (DMEM) containing 50 µg/ml of gentamicin. After 45 min incubation, the cells were washed three times with PBS, followed by the addition of complete DMEM without antibiotics. The cells were then incubated at 37°C in 5% CO_2_ for 72 h. At 0, 24, 48, and 72 h PI, the J774A.1 cells were washed in PBS, lysed by exposure to water for 5 min, and serial dilutions of the lysate were cultured onto BHICB agar to determine the number of viable intracellular bacteria. Because the assays were done on different days and involved slightly different numbers of bacteria and macrophages, the slope and standard deviation of bacterial survival for each 24-h time period was calculated.

### Virulence of *F. tularensis* LVS mutants in mice

Groups of five C57BL/6 mice (Charles River Laboratories, Wilmington, MA) were inoculated by the intranasal (IN) route with a mixture of 5.9×10^3^ CFU/mouse of LVS and 8.0×10^3^ CFU/mouse of LVSΔ1423/1422. All doses were confirmed by viable plate count on BHICB agar. Five days PI, the mice were humanely euthanized with excess carbon dioxide, and the liver, lungs, and spleen were harvested and cultured onto BHICB with or without 8 µg/ml of kanamycin, and incubated at 37°C in 5% CO_2_ for up to 6 days.

In a separate trial, groups of six-week-old female BALB/c mice (Charles River Laboratories, Wilmington, MA) were inoculated with parent LVS or mutant LVSΔ1423/1422 IN or IP, and survival was monitored for six weeks. The doses used for IN inoculations were about 1.2×10^4^ CFU/mouse of LVS or about 1.6×10^4^ CFU of LVSΔ1423/1422. The doses used in IP inoculations for LVS were 41, 262, or 3,375 CFU/mouse, or 1,124, 11,136, and 33,408 CFU of LVSΔ1423/1422. Challenge experiments were done on three different days.

For tissue clearance, BALB/c mice were inoculated IN with 7.9×10^3^ CFU of strain LVS, 5.0×10^4^ CFU of strain LVSΔ1423/1422 (high dose), or 1.1×10^4^ CFU (low dose) of LVSΔ1423/1422. At 2, 4, or 7 days PI, surviving mice were humanely euthanized and the bacteria were cultured from the liver, lungs, and spleen as described above.

### Protective efficacy of *F. tularensis* LVS mutants in mice

BALB/c mice that survived and were healthy for six to seven weeks following IN or IP inoculation with mutant LVSΔ1423/1422 were challenged with 1.4×10^5^ CFU or 7.9×10^3^ CFU, respectively, of LVS IN, and mortality and clinical symptoms were recorded. Four weeks after challenge, surviving mice were humanely euthanized using excess carbon dioxide, and portions of the liver, lungs, and spleen were homogenized and cultured for *F. tularensis*. Following challenge mice were monitored at predetermined intervals and euthanized if they became moribund.

### Statistical analyses

The Student *t* test [61] was used to evaluate the significance in CLC compositional differences between LVS and mutant LVSΔ1423/1422. ANOVA was used for the comparative persistence of *F. tularensis* strains in liver, lungs, and spleen of mice. The chi-squared test with larger contingency tables was used to analyze the protective efficacy of the mutant in mice [61]. For RT-qPCR the Ct of each replicate was used in an unpaired t test between samples obtained from bacteria grown to enhance CLC (grown on Glc-CDMA at 32°C), or minimize CLC expression (grown in BHIC broth at 37°C). Statistical analyses and *P* values were calculated using InStat software (GraphPad, La Jolla, CA).

## Supporting Information

Table S1 Bacterial strains and plasmids used in this study.
(DOCX)

Table S2 DNA primers used for PCR.
(DOCX)

Table S3 List of oligonucleotides used in RT- and qRT-PCR assays.
(DOCX)
