# Supplementary material for: Isolation and Mutagenesis of a Capsule-Like Complex (CLC) from Francisella tularensis, and Contribution of the CLC to F. tularensis Virulence in Mice
Source: PLoS One. 2011 Apr 22;6(4):e19003. doi: 10.1371/journal.pone.0019003 (PMC3081320; doi:10.1371/journal.pone.0019003)
Supplement: Table S1 — Bacterial strains and plasmids used in this study. (DOCX) [file pone.0019003.s001.docx]

**Supporting Information Table S1.** Bacterial strains and plasmids used in this study

| **Bacterial strains** | **Characterisitcs** | **Reference or source** |
| --- | --- | --- |
| *E. coli* DH5α | F^–^ 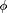80d *lacZ*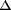M15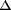(*lacZYA-argF*)*U169 recA1 endA1 hsdR17*(r_K_^–^ m_K_^+^) *phoA supE44* 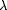^–^ *thi-1gyrA96 relA1* | Invitrogen |
|  |  |  |
| ***F. tularensis*** |  |  |
| LVS | subsp. *holarctica* live vaccine strain | Dr. May Chu, CDC |
| LVS_P10 | LVS passed daily for 10 days in CDMB | This work |
| LVS∆1423/1422 | LVS containing a deletion spanning FTL_1423 to FTL_1422 | This work |
| LVS∆1423/1422_P10 | LVS∆1423/1422 passed daily for 10 days in CDMB | This work |
| LVSΔ1423/1422[1423/1422^+^] | LVS∆1423/1422 containing vector pFTAB-2, expressing the deleted FTL_1423-FTL_1422 region *in trans* | This work |
| LVSΔ1423/1422[1423/1422^+^]_P10 | LVS∆1423/1422[1423/1422^+^] passed 10 times in CDMB | This work |
| WbtI_G191V_ | LVS lacking O-antigen due to a mutation that converts amino acid 191 from a glycine to valine | [11] |
| WbtI_G191V__P17 | WbtI_G191V_ passed daily for 17 days in CDMB | This work |
| WbtI_G191V__P17∆1423/1422 | WbtI_G191V_ containing a deletion spanning FTL_1423-FTL_1422 | This work |
|  |  |  |
| **Plasmids** |  |  |
| pSC-A | PCR Cloning vector, Amp^r^ | Stratagene |
| pSC-1423/1422 | pSC-A containing the regions flanking FTL_1423/FTL_1422 region; Amp^r^ | This work |
| pSC-1423/1422K | pSC-1423/1422 containing the Kan^r^ gene; Amp^r^; Kan^r^ | This work |
| pFNLTP6 | *F. tularensis* shuttle vector; Kan^r^, Amp^r^ | [39] |
| pFTAB-1 | pFNLTP6 containing the entire FTL_1423-FTL_1422 region; Kan^r^, Amp^r^ | This work |
| pFATB-2 | pFTAB-1 containing *cat* (Cm^r^) cassette from pBBR1MCS ligated, Cm^r^, Amp^r^ | This work |
