# Supplementary material for: Isolation and Mutagenesis of a Capsule-Like Complex (CLC) from Francisella tularensis, and Contribution of the CLC to F. tularensis Virulence in Mice
Source: PLoS One. 2011 Apr 22;6(4):e19003. doi: 10.1371/journal.pone.0019003 (PMC3081320; doi:10.1371/journal.pone.0019003)
Supplement: Table S2 — DNA primers used for PCR. (DOCX) [file pone.0019003.s002.docx]

**Supporting Information Table S2.** DNA primers used for PCR.

| **For cloning FTL_1423-FTL_1422 into pSC-A to produce pSC-1423/1422** | |
| --- | --- |
| FTL1424_F_SalI | GCGTCGACTATCAAAGTTGCACCTAG |
| FTL1423_R_StuI | GAAGGCCTGATTTAGCAAAACGATCC |
| FTL1422_F_StuI | AATAGATACGCGCTGGCAAGGC |
| FTL1421_R | ATGTAATCCAACACTCAGATGCAAA |
|  | |
| **For detection of the Kan^r^ gene in recombinant mutant strains** | |
| Kan_CHK_F | AAGTTGGGTAACGCCAGGGTTTTCC |
| Kan_CHK_R | ATTAGGCACCCCAGGCTTTACACTT |
|  | |
| **For verifying the mutation in strain WbtI_G191V_∆1423/1422** | |
| F-FTL-1422 | CAGCAACGGTTGGCTGTAATAAGC |
| R-FTL-1422 | CCTACTTATACAATTTAGGTATAA |
|  | |
| **For cloning FTL_1423-FTL_1422 into pFNLTP6 to produce pFTAB-1** | |
| F-FTL1423-EcoRI | CGGGAATTCGAACATTATAAAATCATCAGA |
| R-FTL1422-XhoI | CGGCTCGAGACTCACTCATTCTCGCCATTT |
|  | |
| **For cloning *cat* gene into pFTAB-1 to produce pFTAB-2** | |
| CAT-Rev | CGGCTGCAGTGACCCGCGACCAGACCACGT |
| CAT-Forw | CGGCTGCAGCCGGTACCCAGCTTTTGTTCC |
