# Supplementary material for: Isolation and Mutagenesis of a Capsule-Like Complex (CLC) from Francisella tularensis, and Contribution of the CLC to F. tularensis Virulence in Mice
Source: PLoS One. 2011 Apr 22;6(4):e19003. doi: 10.1371/journal.pone.0019003 (PMC3081320; doi:10.1371/journal.pone.0019003)
Supplement: Table S3 — List of oligonucleotides used in RT- and qRT-PCR assays. (DOCX) [file pone.0019003.s003.docx]

**Supporting Information Table S3.** List of oligonucleotides used in RT- and qRT-PCR assays.

| **Primer** | **Sequence (5'->3')** |
| --- | --- |
| RT_0800-for | TCAACTATGCTTGGGATGATGCGTT |
| RT_0803-rev | ACGCGCGAACCAGTACCAGC |
| qRT_1428-F | Cgcaacttgatgatttcgttagtaa |
| qRT_1428-R | GCGTTGACCACCCGATAGTC |
| qRT_1426-F | caaATCCCCATATGAGACAAGAAA |
| qRT_1426-R | AATCCTGTCCCAGATCCAAGATC |
| qRT_1424-F | GATGCCTCTACTGATAACTCTCTGG |
| qRT_1424-R | TAAACCCACATTTCTTGCACTAGAT |
| qRT_1423-F | Ctgtaataagcaaataaaagctgct |
| qRT_1423-R | TGCCATTGATGATAACTGTTTTGA |
| qRT_1416-F | GAGCAAAAGCTGATGCTATCGTT |
| qRT_1416-R | CACACAGCGATTGCAAAAGTG |
| qRT_1415-F | GGTCTTGTAGTTGGTCTAGTTTTTG |
| qRT_1415-R | GAGCAAAATATCCAGGTACAACCAT |
| qRT-GAPDH-F | ACTGAGCTTGTTGCTGTCGTATCT |
| qRT-GAPDH-R | CAGCTTTCATCGCAGCATTAA |
